# Shrinkage in the Bayesian analysis of the GGE model: A case study with simulation

**DOI:** 10.1371/journal.pone.0256882

**Published:** 2021-08-30

**Authors:** Luciano Antonio de Oliveira, Carlos Pereira da Silva, Alessandra Querino da Silva, Cristian Tiago Erazo Mendes, Joel Jorge Nuvunga, Joel Augusto Muniz, Júlio Sílvio de Sousa Bueno Filho, Marcio Balestre

**Affiliations:** 1 Faculty of Exact Sciences and Technology (FACET), Federal University of Grande Dourados, Dourados, Mato Grosso do Sul, Brazil; 2 Department of Statistics (DES), Federal University of Lavras, Lavras, Minas Gerais, Brazil; 3 Department of Agriculture, Chibuto College of Business and Entrepreneurship, Eduardo Mondlane University, Chibuto, Gaza, Mozambique; Federal University of Mato Grosso do Sul, BRAZIL

## Abstract

The genotype main effects plus the genotype × environment interaction effects model has been widely used to analyze multi-environmental trials data, especially using a graphical biplot considering the first two principal components of the singular value decomposition of the interaction matrix. Many authors have noted the advantages of applying Bayesian inference in these classes of models to replace the frequentist approach. This results in parsimonious models, and eliminates parameters that would be present in a traditional analysis of bilinear components (frequentist form). This work aims to extend shrinkage methods to estimators of those parameters that composes the multiplicative part of the model, using the maximum entropy principle for prior justification. A Bayesian version (non-shrinkage prior, using conjugacy and large variance) was also used for comparison. The simulated data set had 20 genotypes evaluated across seven environments, in a complete randomized block design with three replications. Cross-validation procedures were conducted to assess the predictive ability of the model and information criteria were used for model selection. A better predictive capacity was found for the model with a shrinkage effect, especially for unorthogonal scenarios in which more genotypes were removed at random. In these cases, however, the best fitted models, as measured by information criteria, were the conjugate flat prior. In addition, the flexibility of the Bayesian method was found, in general, to attribute inference to the parameters of the models which related to the biplot representation. Maximum entropy prior was the more parsimonious, and estimates singular values with a greater contribution to the sum of squares of the genotype + genotype × environmental interaction. Hence, this method enabled the best discrimination of parameters responsible for the existing patterns and the best discarding of the noise than the model assuming non-informative priors for multiplicative parameters.

## Introduction

In multi-environmental trials (MET), it is common to observe differential genotype responses in different environments, and this phenomenon is called genotype × environment interaction (GEI). In the presence of GEI, genotypes with good performance in one environment may perform poorly in another, which imposes difficulties in terms of a wide selection and broad recommendation of superior cultivars. In these situations, it is prudent to group locations (or environments) into relatively homogeneous subgroups. Thus, specific genotypes can be recommended for each of these subgroups [1–3].

Multiplicative (or linear-bilinear) models for fixed effects are widely applicable in the study of patterns of genotype responses between environments [4, 5]. Examples of such models are the main additive effects and multiplicative interaction model (AMMI) and the genotype main effects (G) plus GEI model (G+GEI, or simply GGE), also known as the site regression model (SREG). In AMMI, the main effects and interaction are estimated separately and the bilinear parameter exclusively describes the GEI. In GGE, the main effects of genotypes are not separated from the effect of GEI, and therefore the multiplicative part of the model simultaneously describes the effect of the G+GEI. The bilinear parameters of these models are obtained by Singular Value Decomposition (SVD) of the GEI or G+GEI matrix, which makes it possible to separate noise patterns, thereby approaching high dimensional matrices by matrices with reduced dimensions [2, 6, 7].

Additionally, stability and adaptability can be directly interpreted by inspecting the biplots. The GGE biplot analysis is fundamentally graphical and the first two principal components, obtained by SVD applied to the G+GEI matrix, are central to this procedure. The first principal axis describes the performance (or adaptability) and the second describes the effect of GEI (or stability). The interpretations are based on the properties of the inner product of genotypic and environmental scores in the biplot. Different graphic configurations can be visualized in biplots, which facilitates and enriches the analysis, thereby enabling the identification of subgroups of genotypes (or environments) with similar GEI effects, superior and stable genotypes, and separable mega-environments and specific combinations of genotypes and environments [4, 8–17].

The standard analyses of linear-bilinear models is not flexible enough to deal with unbalanced, non-orthogonal and/or heteroscedastic data. Moreover, no uncertainty measures are presented to the resulting biplot in most applications in the literature [18–20]. Some authors proposed imputation methods and techniques employing preliminary correction of heteroscedasticity and re-scaling, while others proposed more suitable algorithms for weighting scores [21, 22]. Confidence regions in the biplot have also been proposed using asymptotic normality assumption for genotypic and environmental individual scores, or bootstrapping rows and columns from the interaction matrix [19, 20]. This last method has been criticized as it does not preserve the underlying patterns of the interaction [21, 23, 24]. On the contrary, Bayesian methods deal with these situations (unbalanced and/or heteroscedastic data and biplot inference) using proper and conjugate prior specification [25, 26]. Markov chain Monte Carlo methods (MCMC) made it possible to sample from joint posterior distributions and evaluate marginal posterior distributions that would have been unthinkable decades ago. These methods can extend the class of models already implemented in statistical software [27].

The Bayesian method as applied to multiplicative models has been useful in the analysis of data from MET trials. The pioneering work of Viele and Srinivasan [28] and Liu [29], who conducted the fitting process of the AMMI model using a MCMC, ensured that the model parameters are directly sampled and do not violate the model’s inherent restrictions. Crossa et al. [30] and Perez-Elizalde et al. [31], in turn, showed how to incorporate inference to the AMMI-2 biplot by drawing credibility regions to describe the effect of GEI. The literature also presents further development of this method [20, 32–35].

Most contributions in the literature of Bayesian additive-multiplicative models refer to AMMI. Jarquin et al. [36] and Oliveira et al. [37] are the lone exceptions who discuss GGE models. These authors used non-informative prior distributions for all parameters, and especially for the bilinear terms. As the main reason to use non-informative priors is the lack of relevant information about the parameters, it also yields results that resemble maximum likelihood estimators (for AMMI and GGE). However, minimally informative priors using very intuitive and relevant information can be derived from the model choice, which could make for flexible prior specifications and robust final estimates [27, 38, 39].

The strategy of hierarchical prior specification with successive restriction of the parameter domain can be practical and appears very natural in the case of some situations. In our case, it was adopted for the hyperparameters that specify our model dimension (eigenvalues in the SVD). There are a plethora of methods to incorporate prior knowledge. Among the criticism directed at this freedom of choice is that changing the prior specification can imply modifications in the resulting estimates and qualitatively different conclusions. The problem of specifying prior distributions can be solved by using the principle of maximum entropy [40]. For the parameters of multiplicative models (especially singular values), the maximum entropy principle could be used to replace the common practice of defining hyperparameter values to conjugate priors [40, 41]. The maximum entropy principle aims to maximize the “lack of information” that arises from the prior probability distribution to the posterior [42].

The maximum entropy principle provides us with a theoretical justification for conducting scientific inference with less informative priors, which is also beneficial in a situation where information is incomplete or dubious [43–45]. To achieve this goal, we could maximize the entropy [46, 47] from a somewhat restricted choice of prior distributions.

An extension to the Bayesian AMMI was presented in Silva et al. [34] where two-level hierarchical priors were assigned to singular values, with Jeffrey’s priors to the variance components of those parameters. This extension would be analogous to the frequentist shrinkage estimator for the singular values in Cornelius et al. [48] and Cornelius and Crossa [49], thereby respecting the inherent restrictions to singular values. This approach favors obtaining estimates that are restricted to the parametric space and also prevents the use of methods to determine the number of components to be retained in the model to explain GEI. In the study by Silva et al. [34], the estimates of the first singular values are more pronounced, and components related to higher dimensions, with little contribution to explaining variations in the data, are shrunk toward zero.

As already mentioned, the GGE model has a strong graphic appeal, and the first two principal components are essential for the study of genotype responses in environments. Graphical analyses based on properties of the inner product between the singular vectors (genotypic and environmental) in the biplot allow for the identification of genotypes with high yield, wide adaptability (stability), and also suggest the adaptation of cultivars to specific environments, to state the winning genotypes in each of them. In this sense, exploring the analytical properties of the shrinkage estimators for the parameters that describe the G+GEI effect, using Bayesian inference, would be very relevant.

Thus, the current research aims to: i) illustrate how the effects of shrinkage can be extended to the GGE model using maximum entropy prior distributions; ii) use cross-validation procedures to compare the models with and without shrinkage effects, and iii) show the implications and possible interpretations for the bivariate credibility regions incorporated in the biplot representation in a simulated scenario.

## Materials and methods

### Simulated data

We used simulations to generate the dataset for further analysis. The dataset considered a total of 20 genotypes, G = {1,2…,20}, evaluated in seven different environments, E = {1,2,…,7}, assuming a randomized block design with three replications. The main effects were simulated with Gaussian distributions, genotypes (G) ~ *N*(0, 4), environments (E) ~ *N*(0, 4) and blocks (B) ~ N (0,1).

Three distinct response patterns were considered for the GEI: i) {G1, G2, G3, G4, G5} ~ N (0,4), with positive values in the {E1, E2, E3, E4} subgroup and negative values in the {E5, E6, E7} subgroup; ii) {G6, G7, G8, G9, G10} ~ N (0,4), with negative values for {E1, E2, E3, E4} subgroup and positive values in the {E5, E6, E7} subgroup, and iii) {G11, G12,…, G20} ~ N (0,1) for all environments. The subgroup of genotypes formed in i) and ii) are those that have a different response pattern between environments and are called unstable genotypes. The subgroup of genotype iii) corresponds to the subgroup that does not have a different response pattern between environments and is called stable genotype.

The simulated values were organized in a two-way table and corrected for the effects of rows (genotypes) and for the effects of column (environments), thus, constituting the GEI interaction matrix. Each observation in the simulated set was obtained by adding the respective effects of genotypes, environments, blocks, and interactions to a general mean and an error from *N*(0, 6) distribution. Descriptive measures of the simulated data are listed in the S1 Appendix.

### Statistical model

In the GGE model, the vector **y**contains *n* = *v*×*r* phenotypic responses, where *v* is the number of genotypes that are repeated *r* = *b*×*l* in the combinations of blocks (*b* denotes the number of blocks) within locations (*l* denotes the number of locations). This is represented by:

$$y=X_{1}\beta +\overset{t}{\underset{k=1}{\sum }}\lambda_{k}diag(Z\alpha_{k})X_{2}\gamma_{k}+\varepsilon$$ (1)

where **β**_*r*×1_ indicates the vector of the effects of blocks within locations. The terms *λ*_*k*_, **α**_*k*_, and **γ**_*k*_ indicate the singular value and the genotypic and environmental singular vectors related to the *k*th principal component, respectively, where *k* = 1,…,*t* and *t*{*t* = min(*v*−1,*l*)} is the rank of the matrix **GGE**_(*r*×*c*)_. Subsequently, **X**_1_, **X**_2_, and **Z** are design matrices, and the vector **ε**_*n*×1_ contains the effects of experimental errors, with $\varepsilon |\sigma_{e}^{2}∼N_{n}(0,\sigma_{e}^{2}I_{n})$; specifically, $\sigma_{e}^{2}$ is the residual variance, **0**_*n*×1_ the null vector, and **I**_*n*_ the *n* order identity matrix.

The data vector **y**, conditioned on model parameters, is a realization from the following multivariate normal distribution: $y|\beta ,\lambda ,\alpha ,\gamma ,\sigma_{e}^{2}∼N(\mu_{y},I_{n}\sigma_{e}^{2})$, where $\mu_{y}=X_{1}\beta +\sum_{k=1}^{t}\lambda_{k}diag(Z\alpha_{k})X_{2}\gamma_{k}$.

### Assignment of prior information

After defining the model derived from the sampling process (likelihood specification), we assigned prior distributions for all parameters in the model. This step results in a considerable methodological boost from the Bayesian inference as we can quantify the degree of uncertainty (or belief) regarding each of the unobserved variables in the model using probabilistic reasoning [50, 51].

In both compared methods, prior densities were specified as: $\beta |\mu_{\beta },\sigma_{\beta }^{2}∼N(0,\;I10^{8})$; $\lambda_{k}|\mu_{\lambda_{k}},\sigma_{\lambda_{k}}^{2}∼N^{+}(0,\sigma_{\lambda_{k}}^{2})$; **α**_*k*_ ~ spherical uniform in the corrected subspace; **γ**_*k*_ ~ spherical uniform in the corrected subspace; and $p(\sigma_{e}^{2})\propto 1/\sigma_{e}^{2}$ [37]. Note that we chose to specify the hyperparameter for $\sigma_{\beta }^{2}=10^{8}$ to reflect little prior knowledge as it is well justified by Bernardo and Smith [52] and more recently by Zeng et al. [53]. For the residual variance $\sigma_{e}^{2}$ we used Jeffrey’s prior specification, which also reflects lack of knowledge about parameters. This improper prior, however, results in proper full conditional posterior distributions [52, 54], and has no inferential problem as we performed a numerical evaluation of marginal likelihoods [55].

Special attention is paid to the prior density of *λ*_*k*_, which is the positive normal subject to the order relationship *λ*_1_≥⋯≥*λ*_*t*_≥0, with $\mu_{\lambda_{k}}=0$ (*k* = 1,⋯,*t*).

The crucial step of modeling prior knowledge about the variance of singular values model parameters was done by comparing two different specifications:

- BGGE (Bayesian-GGE) model: in this case we chose $\sigma_{\lambda_{k}}^{2}=10^{8}$, as noted by Oliveira et al. [37].
- BGGEE (Bayesian-GGE entropy) model: in this case, the inverted gamma $\sigma_{\lambda_{k}}^{2}∼GI(a,b)$ choice is based on the concept of maximum entropy [40], with a degree of freedom *a* = 1 and scale parameter *b* = 0. This prior is the same as that obtained by Silva [34]. The S2 Appendix provides details on the maximum entropy principle and derivation of this prior distribution.

### Fully conditional posterior densities

From the Bayesian perspective, all inferences are made on joint posterior distribution [56], which is obtained by connecting the information of the likelihood function (according to model 1)

$$L\left(\theta ,\sigma_{e}^{2}|y\right)=p(y|\theta ,\sigma_{e}^{2})=\frac{1}{{\left(2\pi \right)}^{\frac{n}{2}}{\left|I\sigma_{e}^{2}\right)}^{\frac{1}{2}}}\exp\{-\frac{1}{2\sigma_{e}^{2}}{\left(y-\theta \right)}^{⊤}\left(y-\theta \right)\},$$ (2)

together with the priors assumed. Consequently, according to Bayes’ theorem, the joint posterior distribution is as follows:

$$\begin{array}{l} p(\Phi |y)=p(y|\theta ,\sigma_{e}^{2})\propto p(\beta |\mu_{\beta },\sigma_{\beta }^{2})p(\sigma_{e}^{2}|v,u)\times \\ \;\times \prod_{k=1}^{t}p(\lambda_{k}|\mu_{\lambda_{k}},\sigma_{\lambda_{k}}^{2})p(\sigma_{\lambda_{k}}^{2})p(\alpha_{k})p(\gamma_{k}) \end{array}$$ (3)

where $\Phi =(\beta ,\alpha ,\gamma ,\lambda ,\sigma_{e}^{2},\sigma_{\lambda })$, **λ** = (*λ*_1_,⋯,*λ*_*t*_), $\sigma_{\lambda }=(\sigma_{\lambda_{1}}^{2},\cdots ,\sigma_{\lambda_{t}}^{2})$ and *t* = *min*(*g*−1,*e*).

Given the assumptions about hyperparameters of the prior densities, the complete conditional posterior density for each parameter of the GGE model can be obtained by algebraic manipulations concerning the expression (3). They are practically identical to those found in Oliveira et al. [37], except for the prior assumed for the variance of the singular value of the BGGEE. The conditional distributions for the model parameters are as follows:

$$\beta |\ldots ∼N[{\left(X_{1}^{⊤}X_{1}+I\frac{\sigma_{e}^{2}}{\sigma_{\beta }^{2}}\right)}^{-1}X_{1}^{⊤}\left(y-M\right),\;{\left(X_{1}^{⊤}X_{1}\right)}^{-1}\sigma_{e}^{2}],$$ (4)

where $M=\sum_{k=1}^{t}\lambda_{k}{\mathrm{diag}(Z\alpha }_{k}{)X}_{2}\gamma_{k}$;

$$\lambda_{k}|\ldots ∼N^{+}[{\left(\Lambda_{k}^{⊤}\Lambda_{k}+\frac{\sigma_{e}^{2}}{\sigma_{\lambda_{k}}^{2}}\right)}^{-1}\Lambda_{k}^{⊤}M_{k'}^{⊤},{\left(\Lambda_{k}^{⊤}\Lambda_{k}\right)}^{-1}\sigma_{e}^{2}],$$ (5)

where **Λ**_*k*_ = *diag*(**Zα**_*k*_)**X**_2_**γ**_*k*_; $M_{k'}=y-X_{1}\beta -\sum_{k'\neq k}^{t}\lambda_{k'}diag(Z\alpha_{k'})X_{2}\gamma_{k'}$ and *λ*_1_≥…≥*λ*_*t*_≥0.

As already emphasized, the distinction between BGGE and BGGEE is exclusively due to the prior assumption for the hyperparameter $\sigma_{\lambda_{k}}^{2}$. For BGGE, the prior assumption considers the value to be constant and equal to 10^8^ and, therefore, the solutions are equivalent to those obtained by maximum likelihood.

For the BGGEE model, the prior assumption considers uncertainty about the variance of each singular value, and the full conditional posterior distribution for $\sigma_{\lambda_{k}}^{2}$ is given by:

$$p\left(\sigma_{\lambda_{k}}^{2}|\cdots \right)\propto {\left(\sigma_{\lambda_{k}}^{2}\right)}^{-(a+1)-1}exp\{-\frac{1}{2\sigma_{\lambda_{k}}^{2}}(\lambda_{k}^{2}+2b)\},$$ (6)

which corresponds to the nucleus of an Inverse-Gamma density with parameters of scale equal to $\left(\lambda_{k}^{2}+2b\right)$ and degrees of freedom equal to (*a*+1), that is, $\sigma_{\lambda_{k}}^{2}|\cdots ∼GI(a+1,\lambda_{k}^{2}+2b)$. The value of *a* (*a* = 1) is obtained in the algebraic deduction and considering *b* = 0 obtains $\sigma_{\lambda_{k}}^{2}|\cdots ∼GI(2,\;\lambda_{k}^{2})$. Algebraic details are presented in the S3 Appendix.

For the singular vectors, the conditional distributions are given, respectively, by

$$p(\alpha_{k}|\cdots )\propto \exp\{\frac{\lambda_{k}}{\sigma_{e}^{2}}\left[\alpha_{k}^{⊤}\Lambda_{\alpha_{k}}^{⊤}(y-X_{1}\beta )\right]\}$$ (7)

where $\Lambda_{\alpha_{k}}=diag(X_{2}\gamma_{k})Z$, and

$$p(\gamma_{k}|\cdots )\propto \exp\{\frac{\lambda_{k}}{\sigma_{e}^{2}}\left[\gamma_{k}^{⊤}\Lambda_{\gamma_{k}}^{⊤}(y-X_{1}\beta )\right]\}$$ (8)

where $\Lambda_{\gamma_{k}}=diag(Z\alpha_{k})X_{2}$.

The supports of *p*(**α**_*k*_|⋯) and *p*(**γ**_*k*_|⋯) are not trivial due to the restriction of $\alpha_{k}^{⊤}\alpha_{k'}^{}=\gamma_{k}^{⊤}\gamma_{k'}^{}=0$. Thus, sampling is performed by defining auxiliary variables $\alpha_{k}^{*}=H_{k}^{⊤}\alpha_{k}$ and $\gamma_{k}^{*}=D_{k}^{⊤}\gamma_{k}$ (with the correct support) in a corrected subspace. The matrices **H**_*k*_ and **D**_*k*_ have orthogonal columns and are orthogonal to **α**_*k*’_(**α**_*k*’_≠**α**_*k*_) and **γ**_*k*’_(**γ**_*k*’_≠**γ**_*k*_), respectively.

The posterior conditional densities for these variables, in the respective corrected subspaces of dimensions *r*−*p* and *c*−*p* with *p* = *k*−1 for *k* = {1,⋯,*t*}, are von Mises-Fisher (*vMF*) distributions, given by:

$$\alpha_{k}^{*}|\cdots ∼\nu MF(r-p,\frac{c_{k}\lambda_{k}}{\sigma_{e}^{2}},{\tilde{\mu }}_{\alpha_{k}})$$ (9)

with the concentration parameter $c_{k}\lambda_{k}/\sigma_{e}^{2}$, where $c_{k}=\sqrt{{\left(H_{k}^{⊤}\mu_{\alpha_{k}}\right)}^{⊤}H_{k}^{⊤}\mu_{\alpha_{k}}}$ and with directional mean vector resulting in ${\tilde{\mu }}_{\alpha_{k}}=c_{k}^{-1}H_{k}^{⊤}\Lambda_{\alpha_{k}}^{⊤}(y-X\beta )$ and $\mu_{\alpha_{k}}{=\Lambda }_{\alpha_{k}}^{⊤}({y-X}_{1}\beta )$.

$$\gamma_{k}^{*}|\cdots ∼\nu MF(d_{k}-p,\frac{d_{k}\lambda_{k}}{\sigma_{e}^{2}},{\tilde{\mu }}_{\gamma_{k}}),$$ (10)

with $d_{k}\lambda_{k}/\sigma_{e}^{2}$ being the concentration parameter, where $d_{k}=\sqrt{{\left(D_{k}^{⊤}\mu_{\gamma_{k}}\right)}^{⊤}D_{k}^{⊤}\mu_{\gamma_{k}}}$ and with the directional mean vector resulting in ${\tilde{\mu }}_{\gamma_{k}}=d_{k}^{-1}D_{k}^{⊤}\Lambda_{\gamma_{k}}^{⊤}(y-X_{1}\beta )$ and $\mu_{\gamma_{k}}=\Lambda_{\gamma_{k}}^{⊤}(y-X_{1}\beta )$.

In turn, the complete a posteriori conditional density for the residual variance is given by a scaled inverse Chi-square distribution:

$$\sigma_{e}^{2}|\ldots ∼inv-\chi^{2}[n,\frac{{\left(y-\mu_{y}\right)}^{⊤}(y-\mu_{y})}{n}],$$ (11)

with the scaling parameter equal to (**y**−**μ**_**y**_)^⊤^(**y**−**μ**_**y**_)/*n* and the degree of freedom equal to *n*.

### The MCMC sampling and posterior inference

The sampling process was conducted using the Gibbs sampler, with an algorithm described by Oliveira et al. [37]. To obtain the BGGEE, hyperparameters $\sigma_{\lambda_{k}}^{2}$ (*k* = 1,⋯,*t*) sampling was analogous to the scheme used by Silva et al. [34]. The algorithms are presented in the S4 Appendix.

The sampling of singular vectors is not carried out directly, as they must be orthogonal to each other, thereby resulting in support for their posterior densities that are not trivial. Sampling must be performed in a corrected subspace, defining auxiliary variables by orthogonal linear transformation. In this subspace, the variables have no restraints and can be sampled, being back transformed in the correct subspace. The method for carrying out this sampling was first described by Viele and Srinivasan [28].

The length of MCMC chains in each analysis was defined by Raftery and Lewis’ [57] diagnostic criterion (RL) evaluated in a pilot sample size of 4.000. The sample size from the posterior distribution varied following the formula: *N = 4*.*000 J+B*, in which *N* is the final sampling size, *B* is the burn-in parameter as suggested by the RL evaluation or at least *B* = 10.000; J = 20 was used for the thinning parameter, that was the maximum value for the dependency factor (I) in the pilot samples. Final samples has passed both RL diagnostics, with effective sample size greater than 4000 and the I<5 and Heidelberger and Welch’s [58] criterion using α = 5%. Trace plots were also checked to eventually detect possible abnormalities in the posterior sampling. The trace plot consists of a graphic type in which the number of simulations performed for the parameter is represented on the abscissa axis, while the simulated values are represented on the ordinate axis [59]. This graphical representation of the posterior distribution is one method to visually assess whether a chain is in its stationary regime [60, 61].

Estimates such as the posterior means, maximum *a posteriori* (MAP), and 95% highest posterior density credibility intervals (HPD) were obtained from the samples of the joint posterior distribution. HPD were empirically constructed using Chen and Shao’s [62] method, as implemented in the “boa” package from R software [63]. The 95% credible intervals were estimated in the biplot for the first two principal axes with respect to the genotypic and environmental scores $\left(\lambda_{1}^{1/2}\alpha_{i1},\lambda_{1}^{1/2}\alpha_{i2}\right)$ and $\left(\lambda_{1}^{1/2}\gamma_{j1},\lambda_{1}^{1/2}\gamma_{j2}\right)$ with *i* = 1,⋯,20 and *j* = 1,⋯,7, in each sampling step, using Hu and Yang’s [19] method.

### Prediction analysis and model selection

A cross-validation approach was used to assess the predictive ability of each model. We considered three scenarios as function of data removal performed at random, namely: 10%, 33%, and 50% of genotype removal, or 10-fold, 3-fold, and 2-fold, respectively. The predictive ability of the models was quantified by the average predicted residual error sum of squares (PRESS) and phenotypic correlation between the predicted (${\hat{y}}_{ij}$) and the observed (*y*_*ij*_) values (COR). As described in Nuvunga et al. [64], PRESS and COR are calculated by

$$PRESS=\frac{1}{n}\sum_{j=1}^{n}{\left(y_{ij}-{\hat{y}}_{ij}\right)}^{2}$$ (12)

and

$$COR=\frac{\sum_{j=1}^{n}\left({\hat{y}}_{ij}-{\bar{\hat{y}}}_{ij}\right)\left(y_{ij}-{\bar{y}}_{ij}\right)}{\sqrt{\sum_{j=1}^{n}{\left({\hat{y}}_{ij}-{\bar{\hat{y}}}_{ij}\right)}^{2}}\sqrt{\sum_{j=1}^{n}{\left(y_{ij}-{\bar{y}}_{ij}\right)}^{2}}},$$ (13)

where ${\bar{\hat{y}}}_{ij}$ is the mean of the values predicted by the model, ${\bar{y}}_{ij}$ is the mean of the values predicted for validation, and *n* is the number of data removed. As per the PRESS criterion, the lowest value indicates better performance, while in the case of the COR criterion, better performance is indicated by the highest value.

The models were selected according to the number of bilinear components and with the following information criteria: the Bayesian information criterion, or BIC [65]; Akaike’s [66] information criterion, or AIC; and the Akaike-Monte Carlo information criterion, or AICM [55]. In all cases the version of the criteria is presented as “the lower the better”.

Posterior modes for residual variance were worked out for all models adjusted with every possible number *k* (*k* = 1, …, *t*) of bilinear components, where *k* specifies the model dimension (the model with all *t* components fitted will be referred to as the full model). This process was done to estimate influence of model dimension (as a function of *k*) in the residual variance estimates. As the values of the residual variance estimates stabilize from a given number *k* (or dimension *k*), there is no information gain by the inclusion of new dimensions to explain G+GEI.

Information rate (IR) criterion was also evaluated. It is a specific frequentist criteria to separate noise from signal [15] and is given by:

$$IR_{k}=\frac{\lambda_{k}^{2}}{\sum_{k=1}^{t}\lambda_{k}^{2}}t$$ (14)

where *k* = 1,⋯,*t*.

To interpret this criterion, it should be noted that the maximum number of singular values is *t* = *min*(*r*, *c*), which refers to the smaller dimension (number of rows or number of columns). With uncorrelated rows and columns, it follows that the proportion of the total variance explained by each principal component is 1/t. In the presence of correlations, the proportion of variation explained by the first few PC would be greater than 1/t, while for the others it would be smaller. Thus, evaluation of the IR rate allows for a criterion to decide whether the k-axis (PC_k_) should be kept in the interaction model. IR_k_ > 1 implies that the respective PC_k_ summarizes information from more than one variable; therefore, it identifies patterns (or relationships) that should be modeled. On the contrary, if IR_k_ < 1, for some PC_k_, the information is already explained by previous dimensions and could be interpreted as noise; thus this component and the ones after it must be discarded [14, 21].

The entire inference and simulation process was performed using a tailored algorithm in R statistical software [67].

## Results

### Convergence of Markov chains and description of full models

For all parameters from all fitted models we could draw joint posterior samples with good properties as evaluated by Raftery and Lewis and Heidelberg and Welch criteria. All variance components of the models had values for the dependency factor less than five (*I<5*) [57]; they also passed the stationarity test according to Heidelberger and Welch’s criteria [58]. Linear parameters has even better convergence properties confirming theoretical and empirical results in the literature [68]. The S1 Fig depicts trace and density plots of posterior distribution for the complete models’ residual variances, in which all components were retained, just to corroborate the results obtained by the tests.

Table 1 displays the posterior means for the singular values, squared root of the eigenvalue associated with the respective principal component, obtained by the BGGE and BGGEE, according to the number of bilinear terms retained in the adjustment of each model. This table also presents the frequentist estimates (GGE-fixed) obtained from the SVD of the G+GEI matrix. These summaries of the joint posterior distribution illustrate the shrinkage effect of Bayesian posterior estimates, especially for the third singular value for the BGGEE. The first two principal components (PCs) of the GGE-fixed and BGGE explain 90% and 95% of the variance in the G+GEI, respectively. For the BGGEE, the two initial components explain nearly all the variability (>99.9%).

**Table 1:** The posterior means for singular values for the BGGE and BGGEE models as a function of the number of bilinear terms (k = 1,2,^…^, 7).

**BGGE Model**							
*K*	*λ*_1_	*λ*_2_	*λ*_3_	*λ*_4_	*λ*_5_	*λ*_6_	*λ*_7_
1	46.29						
2	46.49	16.17					
3	46.52	16.39	9.74				
4	46.53	16.44	9.84	5.23			
5	46.57	16.51	9.90	5.41	2.12		
6	46.61	16.48	9.92	5.43	2.14	0.87	
7	46.56	16.46	9.91	5.40	2.13	0.86	0.42
**BGGEE Model**							
*K*	*λ*_1_	*λ*_2_	*λ*_3_	*λ*_4_	*λ*_5_	*λ*_6_	*λ*_7_
1	46.07						
2	46.29	15.69					
3	46.32	15.66	<0.001				
4	46.25	15.69	<0.001	<0.001			
5	46.27	15.65	<0.001	<0.001	<0.001		
6	46.31	15.75	<0.001	<0.001	<0.001	<<0.001	
7	46.31	15.69	<0.001	<0.001	<0.001	<<0.001	<<0.001
**GGE-fixed**	47.04	17.72	11.8	8.85	6.79	4.53	2.70

BGGE, Bayesian-GGE; BGGEE, Bayesian-GGE entropy; A<<B implies A<0.01B.

**Fig 1:**
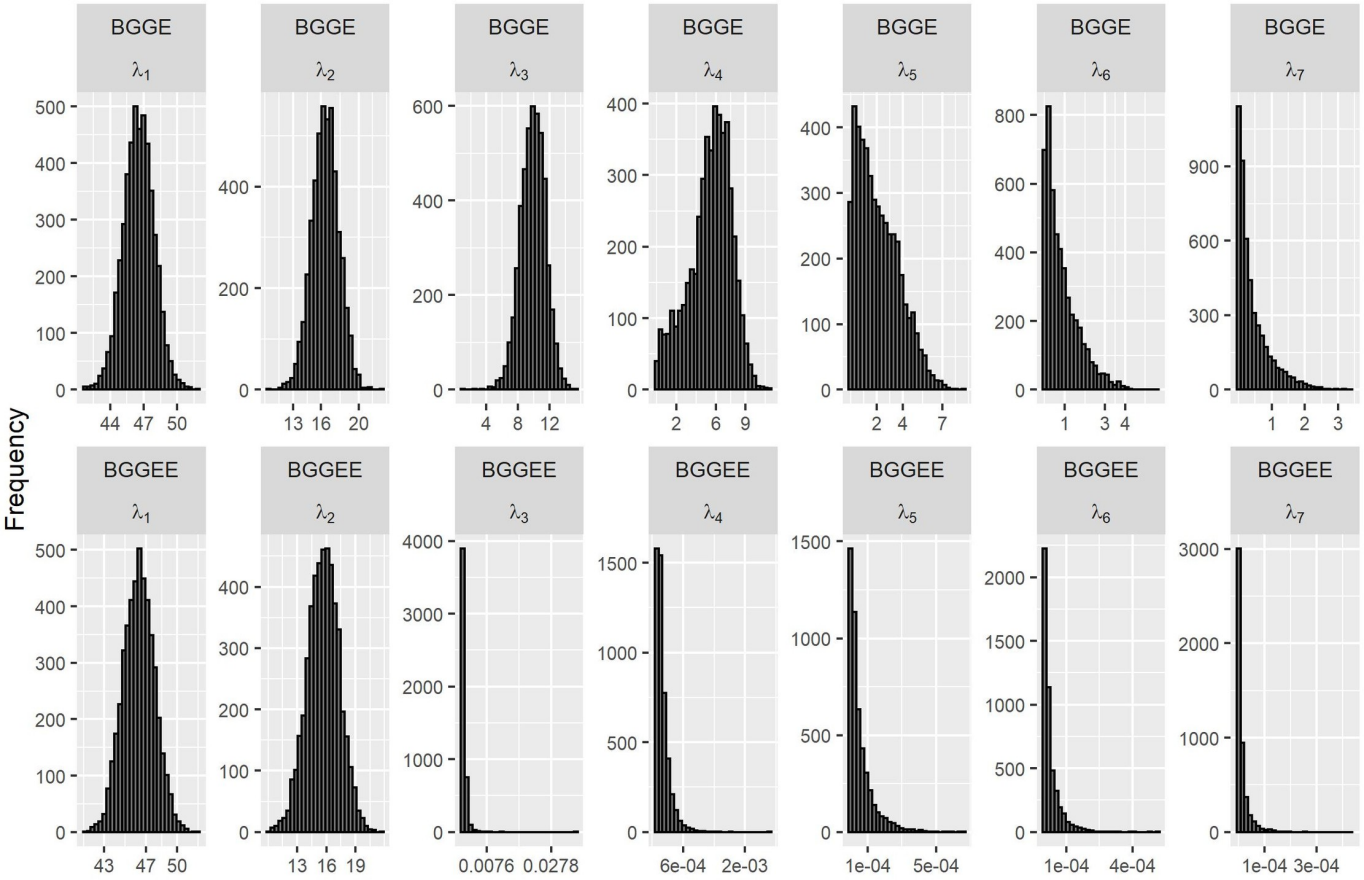
Histograms of the posterior marginal distributions of singular values, considering models of the complete dimensions for the proposed BGGE and BGGEE models.

The histograms (Fig 1) show the posterior distributions of the singular values for BGGE and BGGEE models retaining all possible dimensions (hereinafter called full models). The distributions for the first singular values are approximately symmetric (Gaussian) and as we observed a departure from the first singular values, the distributions of the values became more and more skewed to the right. This finding is clearer for BGGEE and for *λ*_3_, in which the mean, mode, and median are very close to zero, indicating that this one and the higher order components do little to explain the variation of the interaction.

### Prediction analysis: Correlation and PRESS

Table 2 presents the result means the analysis of cross-validation with correlations (COR) and PRESS for each of the proposed three scenarios (*k-fold*). The models are nearly indistinguishable for both criteria evaluated at the 10% level of random data removal. However, the BGGEE model exhibits substantial advantages when removing more data (Table 2). In the S1 Table individual correlations and PRESS values for each *k-fold* (*k* = 10, 3, 2) are presented with the respective standard deviation values.

**Table 2:** Mean values of COR and PRESS for the BGGE and BGGEE models in three random, unbalanced scenarios.

	BGGE Model		BGGEE Model	
Level (%)	COR	PRESS	COR	PRESS
10	0.78	9.13	0.78	8.82
33	0.58	14.46	0.64	13.34
50	0.46	31.11	0.71	10.69

BGGE, Bayesian-GGE; BGGEE, Bayesian-GGE entropy; COR, correlation between the predicted and the observed values; PRESS, average predicted residual error sum of squares.

### Selection of models using information criteria

**Fig 2:**
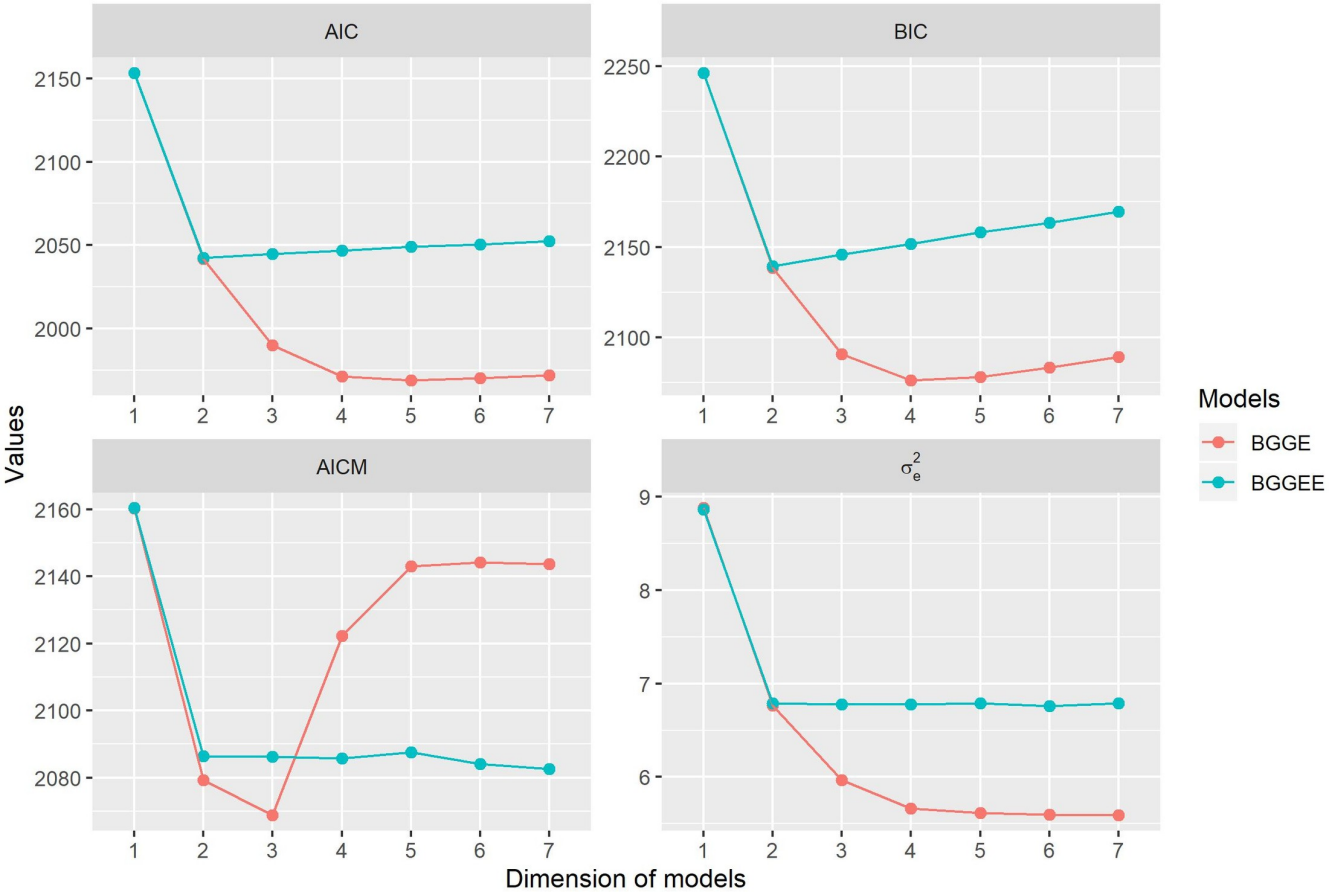
Results of the AIC, BIC and AICM and residual variance $\sigma_{e}^{2}$, calculated for the Bayesian-GGE (BGGE-*k*) and Bayesian-GGE entropy (BGGEE-*k*) family of models for *k* = 1,…, 7.

Fig 2 displays the results obtained using the AIC, BIC, and AICM criteria, as well as the posterior mean for residual variance $\left(\sigma_{e}^{2}\right)$. The BGGEE model retaining two singular values had the best fit for both BIC and AIC. Additionally, there is practically no difference between model dimensions using the AICM criterion. Note that increasing the model dimension when using this criterion showed no change in the residual variance for the BGGEE-2, which makes it the most appropriate choice given the principle of parsimony. For BGGE, different criteria result in different choices of the optimal number of dimensions, the result was three, four, and five bilinear components to be retained using the AICM, BIC, and AIM criteria, respectively.

The IR is typically included in GGE approaches that address fixed effects and quantifies each axis’ contributions to explain the data [15]. It is also a criterion to separate noise patterns and size reduction.

The S2 Fig presents the posterior means and regions of HPD credibility for the information rates in the Bayesian models. Only the first PC stands out, in that IR>1, indicating that the other PCs would not be informative. Regarding the frequentist GGE model, IR values of 5.50 and 0.78 were obtained for PC1 and PC2, respectively. The IR values for subsequent PCs would be even lower due to the properties of principal component analysis.

### Inferences from selected models

Inferences for the bilinear parameters and biplot analysis are presented only for the best models (AICM criterion): the BGGE-3 and the BGGEE-2. Table 3 presents the posterior means and HPD credibility intervals for singular values and variance components for these models; no major discrepancies exist between the estimates for the first two singular values of the two models under analysis. The posterior mean of the BGGE error variance is slightly lower as the HPD region does not include the mean for the BGGEE error variance (despite the intersection between the 95% credibility regions).

**Table 3:** Summaries of posterior distributions for singular values and variance components for the BGGE and BGGEE models.

Model	Parameter	Mean	Sd	95% HPD credibility intervals	
				LL	UL
	*λ*_1_	46.56	1.36	43.95	49.27
**BGGE**	*λ*_2_	16.45	1.43	13.67	19.21
	*λ*_3_	9.91	1.57	6.72	12.83
	$\sigma_{e}^{2}$	5.59	0.49	4.68	6.60
	*λ*_1_	46.31	1.50	43.34	49.17
**BGGEE**	*λ*_2_	15.69	1.64	12.52	18.92
	$\sigma_{\lambda_{1}}^{2}$	260.95	9584.96	132.68	6534.63
	$\sigma_{\lambda_{2}}^{2}$	60.68	1107.23	10.91	737.35
	$\sigma_{e}^{2}$	6.79	0.51	5.79	7.80

BGGE, Bayesian-GGE; BGGEE, Bayesian-GGE entropy; Sd, standard deviation; HPD; highest posterior density credibility intervals; LL, lower limit; UL, upper limit.

**Fig 3:**
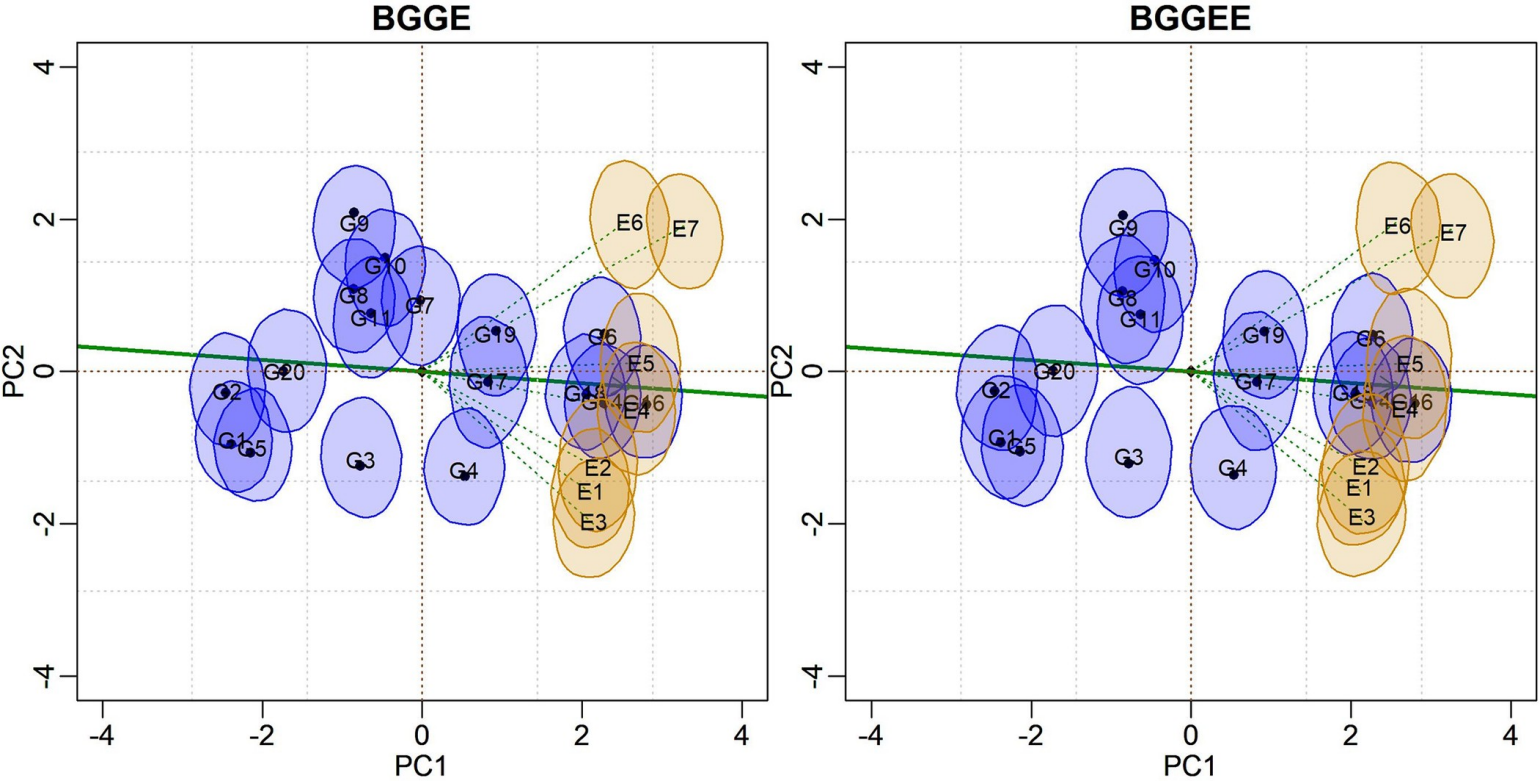
Biplots with 95% credible bivariate regions for the BGGE (Bayesian-GGE) and BGGEE (Bayesian-GGE entropy) models’ genotypic and environmental scores.

Means and bivariate credibility regions based on joint posterior distribution are incorporated into the biplot representation (Fig 3). This graph illustrates the “representativeness versus discrimination” graphic configuration [13]. Environmental vectors are included to allow for an interpretation of the angles between environments as well as their discriminative capacity. An additional average environment axis (AEA) was drawn from the origin of the biplot passing through the environment’s midpoint to indicate its representativeness relative to the target environment. It was observed that environments E4 and E5 were the most representative of the macro-environment in question. Environments E3, E6, and E7 are farther from the origin, with longer vectors and greater discriminative capacity.

The patterns displayed in both biplots are similar, but the G7 in the BGGE biplot is not stable (the credible region does not include the origin). This model allowed a crisper separation between some subgroups of genotypes and environments. The two environmental subgroups {E6, E7} and {E1, E2, E3} are clearly separable, as they are located in different quadrants. Environments E4 and E5 have credibility regions dispersed along the first and fourth quadrants.

Biplot visualization allows for the identification of genotypes with better performance (above the general mean). They are positioned to the right of PC1 (or the AEA). Those with responses below average are positioned to the left. Genotypes with bivariate regions encompassing the biplot’s origin constitute another subgroup in which yields do not statistically differ from the general mean, and the interaction effect is not relevant. Those genotypes were not plotted to simplify interpretations.

However, this global analysis is typically conducted in a preliminary sense. The recommendation is that evaluations of environments, as well as the selection and recommendations of genotypes be carried out within each mega-environment [1, 14]. Two mega-environments may exist—MEGA1 = {E1, E2, E3, E4} and MEGA2 = {E5, E6, E7}—although overlaps occur. It should be noted that we use the term “mega-environment” for didactic purposes, as the proper environmental standard must be established by several years of experimentation. After mega-environment identification, further analyses were conducted for each, using BGGE and BGGEE models.

#### The BGGE model

**Fig 4:**
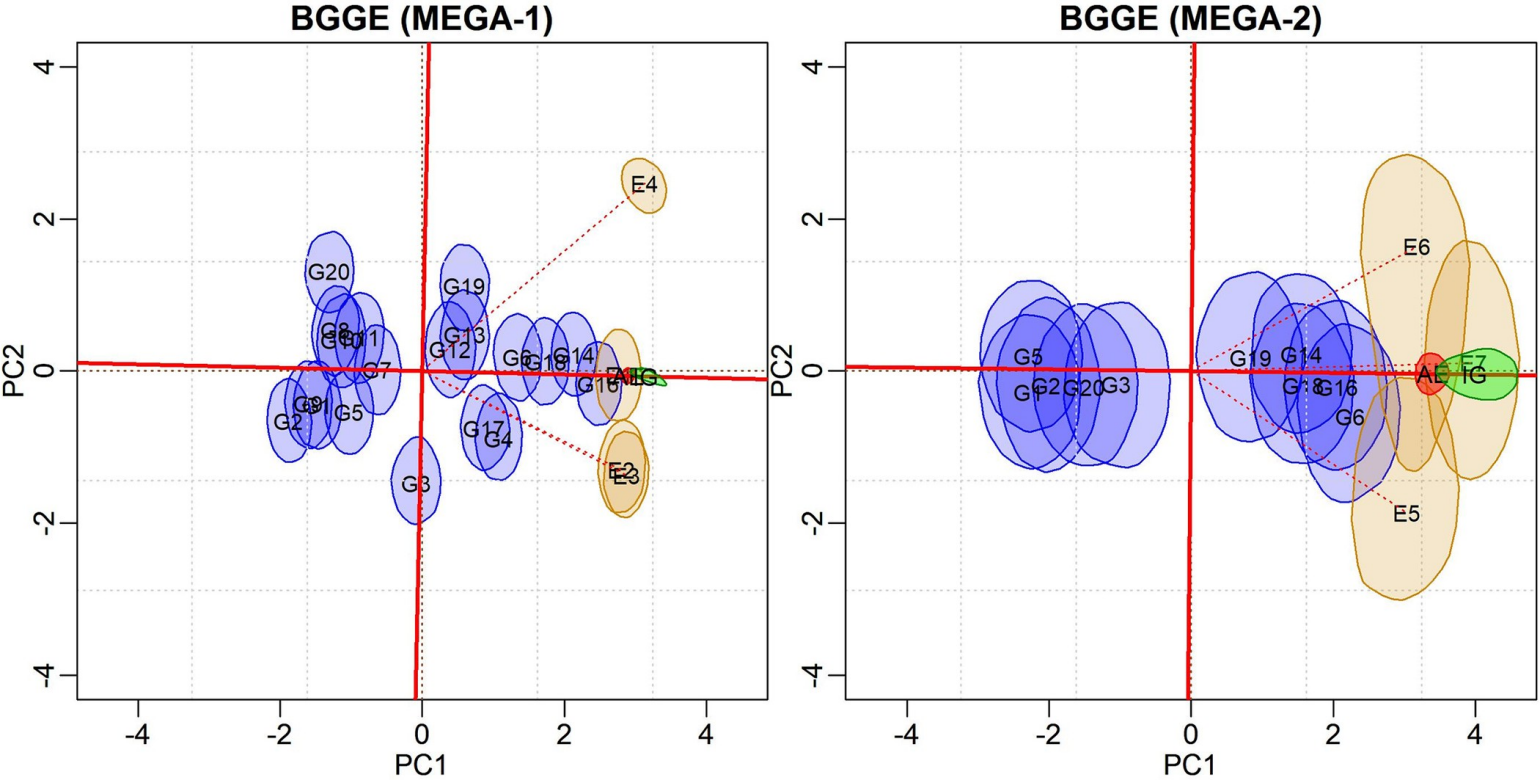
Biplot for the BGGE (Bayesian-GGE) model with 95% credible regions, including the genotypic and environmental scores for the average environment (red) and ideal genotype (green) for the two defined mega-environments.

The configuration in Fig 4 is similar to that presented for the target environment (Fig 3). However, the average environment and ideal genotype were inserted, with their respective regions of bivariate credibility. This graphic form illustrates the “mean versus stability” pattern. Note that there is another axis through the origin but orthogonal to AEA. Genotypes that are farthest from the origin of this axis are those that contribute most to the interaction.

From the biplots in Fig 4 we identify MEGA-1 as the most complex, as the E4 environment’s vector is nearly orthogonal to the {E2, E3} subgroup vector. The E1 environment is the most representative of this mega-environment, as its vector forms an acute angle with the AEA axis. Alternatively, the E4 environment has the greatest capacity for discriminating genotypes, although it is the least representative of this mega-environment. If the objective of this study was to select test sites, only one of the E2 or E3 environments would have been selected, as they offer the same information. The G16 genotype would be the closest to the ideal for this mega-environment, followed by G14, with a region of overlapping credibility.

The MEGA-2 environment is simpler, despite a greater dispersion of its credible regions. For this mega-environment, E7 is the most representative and has the greatest discriminative capacity. It was not possible to highlight a single genotype with superior yield for MEGA-2, and a subgroup of genotypes {G6, G16, G14, G18, G19} have overlapping credibility regions for the distance to the ideal genotype.

#### The BGGEE model

**Fig 5:**
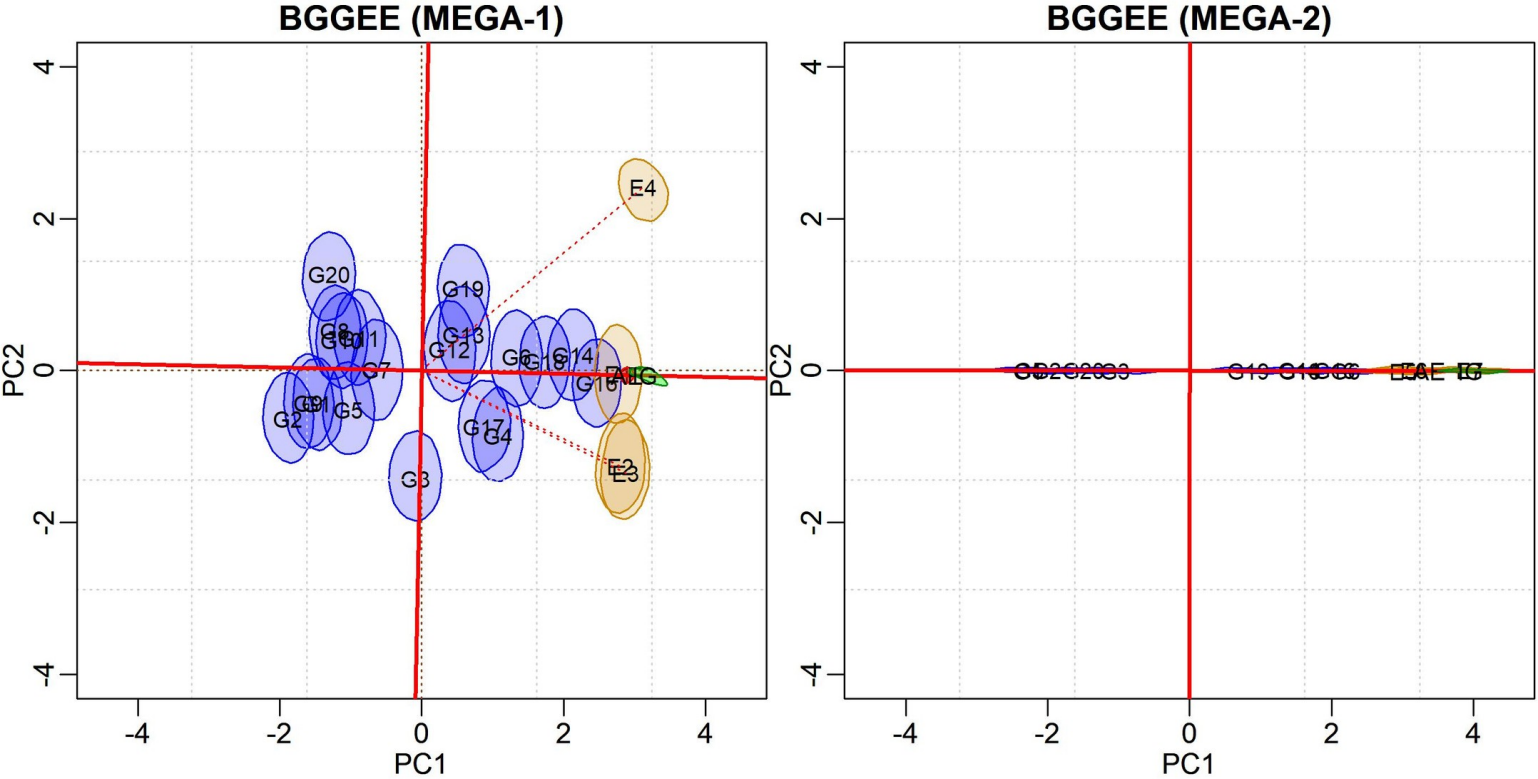
Biplot for the BGGEE (Bayesian-GGE entropy) model with 95% credible regions, including the genotypic and environmental scores for the average environment (red) and ideal genotype (green) for to the two defined mega-environments.

Fig 5 depicts biplots for the mega-environments according to the BGGEE model. As the pattern in MEGA-1 is similar to that observed for the BGGE model, the same conclusions follow. Regarding the MEGA-2, the second singular value has shrunk to near zero. This result implies only one principal axis is needed to explain the G+GEI pattern. Consequently, this mega-environment is more homogeneous and the GEI has drastically decreased. It is possible to observe a subgroup of genotypes with overlapping credible regions. Among them, the G6 and G16 genotypes exhibit the highest means.

Table 4 presents each environment’s correlation with the average environment and its HPD regions. This correlation is approximated by the cosine of the angle formed with the average environment. Note that no prior literature has presented credibility regions (or confidence limits) for this specific correlation as such an estimate would be difficult to deduce or approximate using fiducial or pure frequentist methods.

**Table 4:** Summaries for the posterior distribution of inner products for environments and average environment vectors.

Model	Mega	Env.	Mean	Median	Sd	LL	UL
**BGGE**	1	E1	0.99	0.99	0.006	0.98	1.00
		E2	0.91	0.92	0.029	0.86	0.97
		E3	0.91	0.91	0.028	0.85	0.96
		E4	0.77	0.77	0.025	0.72	0.82
	2	E5	0.85	0.85	0.076	0.73	1.00
		E6	0.87	0.87	0.072	0.75	1.00
		E7	0.99	0.99	0.019	0.95	1.00
**BGGEE**	1	E1	0.99	0.99	0.006	0.98	1.00
		E2	0.92	0.92	0.032	0.86	0.98
		E3	0.91	0.91	0.031	0.85	0.97
		E4	0.78	0.78	0.027	0.73	0.83
	2	E5	0.99	1.00	<<0.001	0.99	1.00
		E6	0.99	1.00	<<0.001	0.99	1.00
		E7	1.00	1.00	<<0.001	0.99	1.00

BGGE, Bayesian-GGE; BGGEE, Bayesian-GGE entropy; Sd, standard deviation; LL, lower limit; UL, upper limit; Env., environment; Mega, mega-environments; A<<B implies A<0.01B.

**Fig 6:**
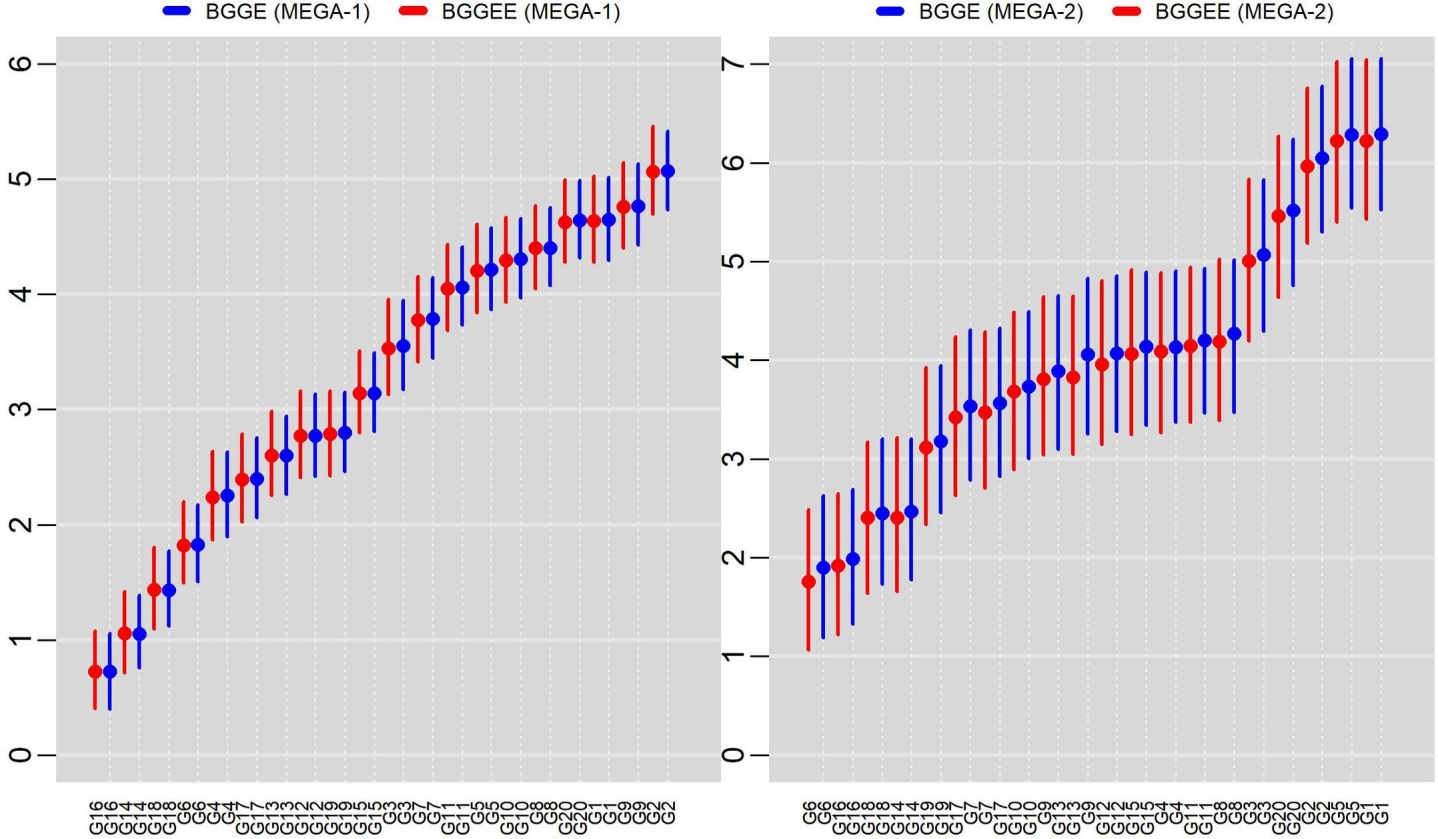
Mean scores and 95% HPD credibility intervals for distances of the genotypes to the ideal genotype in each mega-environment using the BGGE (Bayesian-GGE) and BGGEE (Bayesian-GGE entropy) models.

Fig 6 depicts the posterior means of the distances from each genotype to the ideal, and respective HPD regions for each mega-environment, for both methods. These graphs display the distances to the ideal genotype inferred for G16 and G14 in the first mega-environment, and there is no relevant difference. G16 has marginally the smallest distance to the ideal among all genotypes. In the second mega-environment, G6 and G16 are the closest to the ideal genotype, although their credibility regions overlap with the {G14, G18, G19} subgroup for the two models in question.

The S3–S5 Figs illustrate the “who won where” configuration as a polygon with vertices (genotypes) furthest from the biplot’s origin. In this representation, orthogonal lines to the sides of these polygons pass through the origin, such that the genotypes represented at the vertices have higher yields in environments in the same sector [69].

## Discussion

As observed in our results, BGGE produces some shrinkage if compared to the method of ordinary least squares estimators (OLS). However, this is a common feature of Bayesian methods and the prior probability distribution specification for parameters. BGGEE, in contrast, is similar to the fixed effects model described by Cornelius and Crossa [49] and Cornelius et al. [48], who used a minimum mean square error (MMSE) estimator. The authors emphasized that their method is restricted to balanced homoscedastic cases. Moreover, the two-step procedure they used implies slight changes in the resulting model, violating order restrictions in the singular values. This needs to be corrected by *ad hoc* restrictions to yield final estimates.

The method presented here is sufficiently flexible to deal with missing data and heteroscedasticity, as well as to incorporate other information into the analysis (in the form of *a priori* distributions). In addition, the MCMC sampling of conditional distributions is carried out with correct supports to preserve the relations of the order of singular values and do not violate model restrictions, as shown by Viele and Srinivasan [28].

In our analyses, we note the greater shrinkage after the second singular value estimates that occur in the BGGEE as compared to BGGE (Table 1). This result implies that the first two components described most of the GEI variability and the remaining singular values would not be relevant to interpret these data. A similar effect was observed by Silva et al. [34], who also used specific priors to variance components associated with singular values (Bayesian AMMI). As GGE and AMMI are parametrically different, despite belonging to the same class of models based on SVD, they have different interpretations to scores and singular values. Taking this into account, we would expect a similar behavior among some estimates from both models if the same data were used, but a very different interpretation of biplots and other summaries.

The information criteria did not always point to the same model as the best; however, we determined BGGEE-2 as ideal. In this model, the AICM values (Fig 2) from the second component stabilize and the singular values shrinks to zero, thereby not changing the response pattern (Table 1). Our choice for the AICM criterion follows Silva’s [70] argument that the criterion results in more parsimonious, robust models given the different prior distributions. If the study aims to infer other parameters of interest—such as genotypic predictions, environmental effects, or biplots, among others—the model with all bilinear terms or the with two bilinear terms are equivalent. In other words, the model selection would not be a necessary step in the analysis. Using the IR (S2 Fig) we also conclude that only the first axis would be informative for our example.

More expressive shrinkage effects were not observed for the first two components of the BGGEE relative to the SVD solutions for the GGE fixed model; specifically, these are the truly important dimensions in our analysis.

Consequent to the discussion so far, the prediction regarding the BGGEE model was found consistently superior to that of the BGGE, particularly in the case of severe unbalance (Table 2). This aspect highlights the BGGEE’s superior ability to capture patterns and discard noise. It is noteworthy, however, that the best prediction model is not necessarily the one with the best adjustment (assessed by information criteria) as observed in our analyses. Nevertheless, it is worth noting that the first two principal components always explain more of the variability of the data. In addition, the pattern of interaction in the data set that we analyzed is not very complex and this fact became clear based on the IR values, including for the frequentist version of GGE. Based on the finding, the BGGEE model is a more reasonable choice. It is common for a certain method to produce better performance than another depending on the evaluation criteria, as highlighted by Wolpert and Macready (No Free Lunch Theorem) [71]; the high performance of some algorithms in a class of problems is compensated by their low performance in another class.

One of the main criticisms of conventional GGE analysis is that, in most applications, no measure of uncertainty is added to the genotypic and environmental scores in the biplot, although there are some methods for doing so [18, 23, 72, 73]. Bayesian modeling, in turn, offers a flexible parametric procedure for biplot inference based on the posterior joint distribution, as exemplified here. In addition, we incorporated common settings into the biplot, such as the “who won where” pattern and the “average-environment axis”, as well as credible regions for the average environment and the ideal genotype. Inferences about these quantities have not yet been presented in Bayesian versions of the multiplicative models and it is difficult to implement them in the fixed versions of the GGE model [21].

We also showed that the proposed method is flexible enough to associate uncertainty with other parameters of interest, such as the correlations between environmental scores—approximated by the cosine of their angles—and the distances of each genotype to the ideal genotype. In these cases, point estimates were always presented in standard biplot analysis [21]. Similarly, an inference about any other parameter functions can be incorporated into the GGE biplot settings from the samples of the joint posterior distribution.

A common criticism of Bayesian methods is their sensitivity to specifications of prior distributions, which can lead to significantly different results for the same likelihood. Eliciting prior distributions is very different from deriving them from the maximum entropy principle [40, 41]. We argue that this step virtually solves the subjectivity problem, as it avoids ambiguities in choosing a distribution based on arbitrary decisions.

Silva et al. [34] used Jeffrey’s prior for the variance of the singular value and obtained the shrinkage effect of the estimates, but this choice resulted in a posteriori scaled inverse chi-squared conditional densities with one degree of freedom; as a consequence, the marginal posterior distributions were improper. To address this problem, they used the extended prior specifications from Ter Braak et al. [74]. A drawback of using this correction strategy is that the marginal posterior distributions may be bimodal. In our approach of using the maximum entropy principle for prior specification, such inappropriate or bimodal posteriors did not occur. This method was suggested but not implemented by Silva [70] in the context of the AMMI model.

Bayesian inference using the maximum entropy principle to specify prior distributions results in a more flexible GGE model. This model specification has a significant advantage over fixed-effects methods and its restrictive assumptions. In this sense, the method presented here is promising.

## Conclusions and future research

The Bayesian version of the GGE using the maximum entropy principle to prior specification made it possible to draw inferences that are marginal o the model dimension. With this specification, the full model and models with just two bilinear terms are almost the same (similar biplots, predictions, error variances and other summaries). However, model selection is not a required step; it also avoids the sampling problems observed with Jeffrey’s priors for the variance components of bilinear terms, thereby resulting in proper and uni-modal marginal posterior distributions. The maximum entropy prior allows users to identify a clearer pattern and more efficiently discard noise than other prior specifications in the literature.

Bayesian GGE inference on genotypic and environmental scores in biplot includes posterior approximations for their credibility regions and also for the ideal genotype, which is a novel result for Bayesian or frequentist methods.

Despite its advantages, Bayesian GGE implementation is relatively difficult without suitable software. Other important practical developments that need to be tackled are generalizations for heterogeneous variance and non-continuous response variables. Those aspects can be the focus of future investigation.

## Supporting information

S1 Fig Graph of the trace for variance components. (PDF)

S2 Fig Credibility region for the information rate (IR). (PDF)

S3 Fig Default “who won where” to the target environment. (PDF)

S4 Fig Default “who won where” for mega-environments second BGGE. (PDF)

S5 Fig Default “who won where” for mega-environments second BGGEE. (PDF)

S1 Table Individual COR and PRESS. (PDF)

S1 Appendix Descriptive summaries of simulated data. (PDF)

S2 Appendix Maximum entropy prior. (PDF)

S3 Appendix Posterior distribution for variance components. (PDF)

S4 Appendix Algorithm for MCMC sampling. (PDF)
