# Supplementary figures and images for: Shrinkage in the Bayesian analysis of the GGE model: A case study with simulation
Source: PLoS One. 2021 Aug 30;16(8):e0256882. doi: 10.1371/journal.pone.0256882 (PMC8405011; doi:10.1371/journal.pone.0256882)

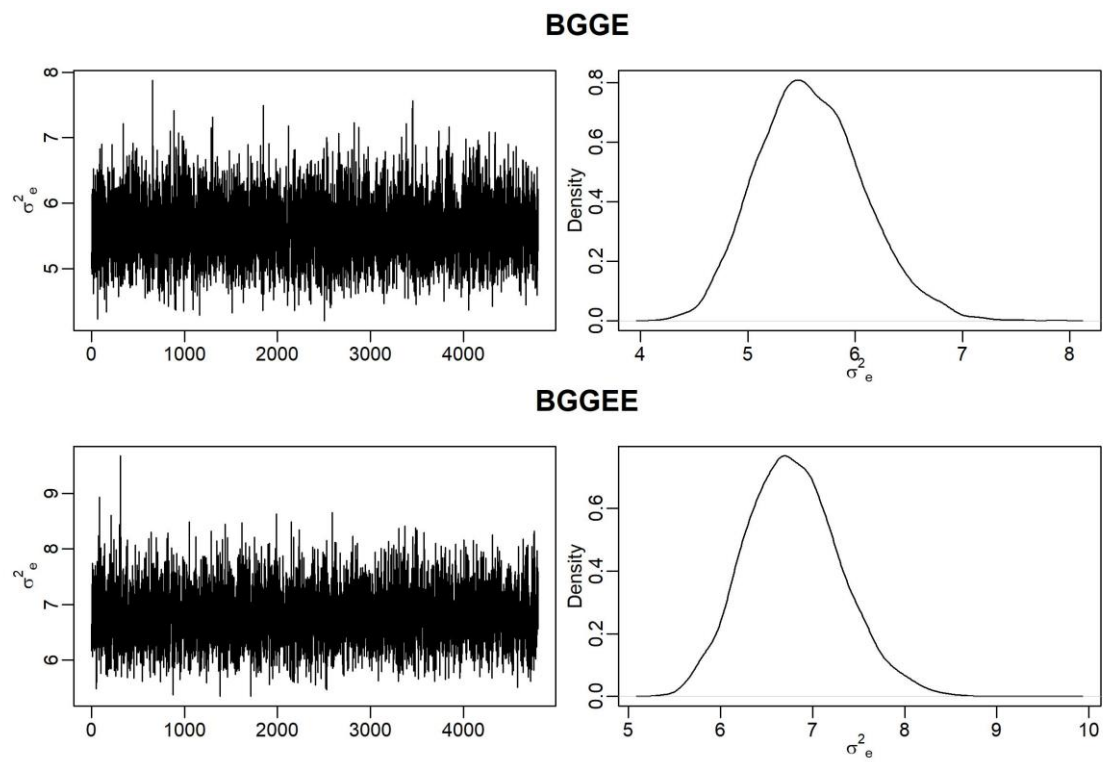

S1 Fig. Response trace and densities graph for the residual variance in the BGGE and BGGEE models.

Supplement: S1 Fig — (PDF) [file pone.0256882.s001.pdf]
