# Supplementary material for: Shrinkage in the Bayesian analysis of the GGE model: A case study with simulation
Source: PLoS One. 2021 Aug 30;16(8):e0256882. doi: 10.1371/journal.pone.0256882 (PMC8405011; doi:10.1371/journal.pone.0256882)
Supplement: S2 Fig — (PDF) [file pone.0256882.s002.pdf]

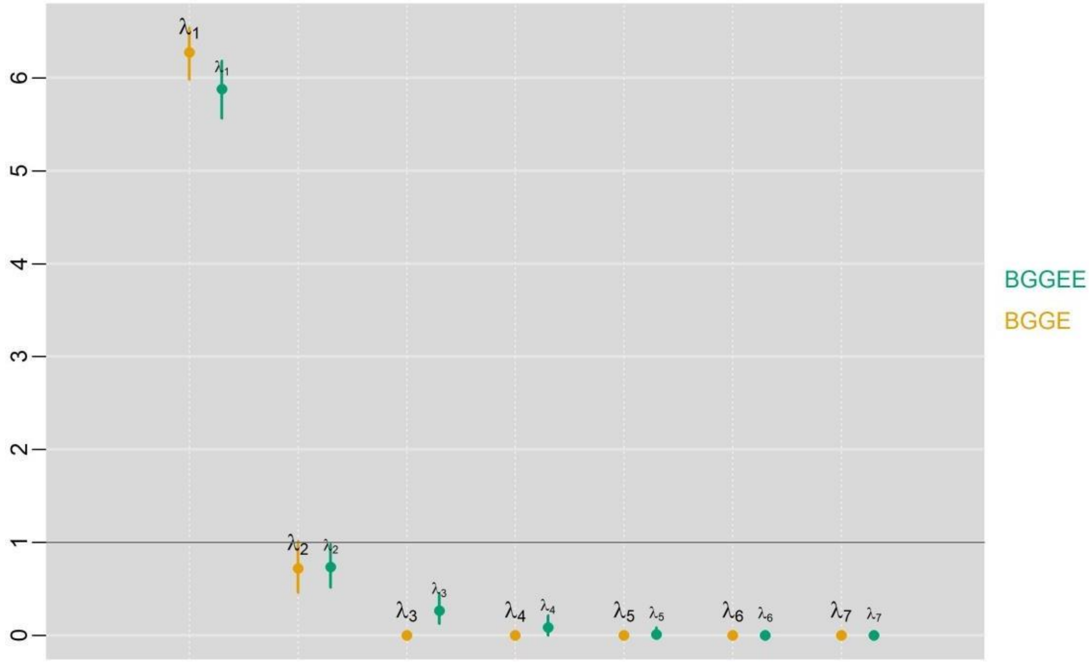

S2 Fig. The posterior means and 95% highest posterior density credibility intervals (HPD) for the information rate (IR), referring to the BGGE and BGGEE models' main components.
