# Supplementary material for: Shrinkage in the Bayesian analysis of the GGE model: A case study with simulation
Source: PLoS One. 2021 Aug 30;16(8):e0256882. doi: 10.1371/journal.pone.0256882 (PMC8405011; doi:10.1371/journal.pone.0256882)
Supplement: S5 Fig — (PDF) [file pone.0256882.s005.pdf]

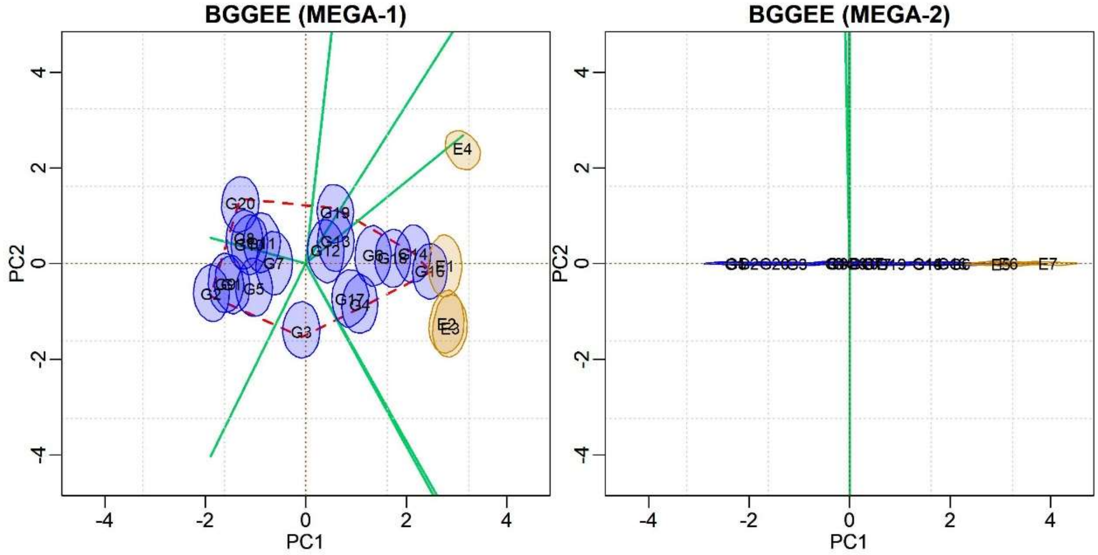

S5 Fig. The GGE biplot and 95% credible regions for the genotypic and environmental scores, showing the “who won where” graphic formulation for the two mega-environments defined according to the BGGEE model.
