# Supplementary material for: Shrinkage in the Bayesian analysis of the GGE model: A case study with simulation
Source: PLoS One. 2021 Aug 30;16(8):e0256882. doi: 10.1371/journal.pone.0256882 (PMC8405011; doi:10.1371/journal.pone.0256882)
Supplement: S1 Table — (PDF) [file pone.0256882.s006.pdf]

S1 Table. Individual COR and PRESS values calculated for the three random unbalanced scenarios (RUS) with simulated genotypes.

BGGE				BGGEE			
Level (%)	Unb.	COR	PRESS	Level (%)	Unb.	COR	PRESS
10	1	0.91	6.37	10	1	0.86	7.27
10	2	0.89	5.20	10	2	0.90	5.12
10	3	0.72	7.00	10	3	0.72	7.08
10	4	0.72	6.03	10	4	0.72	5.97
10	5	0.87	9.00	10	5	0.90	8.24
10	6	0.89	8.58	10	6	0.90	7.65
10	7	0.61	20.82	10	7	0.65	19.66
10	8	0.80	9.51	10	8	0.80	8.87
10	9	0.90	5.37	10	9	0.91	5.09
10	10	0.49	13.37	10	10	0.49	13.22
Mean	-	0.78	9.13	Mean	-	0.78	8.82
Sd	-	0.14	4.79	Sd	-	0.14	4.47
33	1	0.44	15.64	33	1	0.53	14.42
33	2	0.68	13.30	33	2	0.73	12.37
33	3	0.61	14.43	33	3	0.66	13.24
Mean	-	0.58	14.46	Mean	-	0.64	13.34
Sd	-	0.12	1.17	Sd	-	0.10	1.03
50	1	0.67	15.12	50	1	0.68	11.09
50	2	0.24	47.07	50	2	0.74	10.29
Mean	-	0.46	31.11	Mean	-	0.71	10.69
Sd	-	0.31	22.57	Sd	-	0.04	0.56

*Unb. = unbalanced. Sd = standard deviation.
