# Supplementary material for: Shrinkage in the Bayesian analysis of the GGE model: A case study with simulation
Source: PLoS One. 2021 Aug 30;16(8):e0256882. doi: 10.1371/journal.pone.0256882 (PMC8405011; doi:10.1371/journal.pone.0256882)
Supplement: S1 Appendix — (PDF) [file pone.0256882.s007.pdf]

S1 Appendix

Summary of the data and biplot representation for the GGE model of fixed effects.

S1.1 Table - Statistical summary of the data concerning the test environments.

Env.	Mean	Sd	Min	Max
1	15.0713	3.7573	8.2799	21.9696
2	15.1334	4.1278	7.7940	25.5150
3	14.0747	4.1840	6.2905	22.0656
4	15.1637	4.8655	6.1017	26.2763
5	14.5151	5.1857	4.5477	25.6693
6	14.1536	5.4468	-0.5273	24.1450
7	13.9495	6.5202	0.0337	26.1846

S1.2 Table - Means of the genotypes in the test environments.

	E1	E2	E3	E4	E5	E6	E7
G1	11.1799	10.3461	11.5479	9.1445	8.6681	5.3879	4.0222
G2	10.3088	10.7106	9.3724	7.1871	7.5882	4.9016	7.5799
G3	15.8041	17.0865	15.5842	10.4583	11.9654	9.8855	9.4592
G4	17.6844	18.8420	19.5666	16.2695	15.7018	13.9623	12.0763
G5	12.8506	13.1402	11.3696	10.1644	5.9950	7.0502	4.7733
G6	18.4263	17.7218	18.8767	20.0726	23.7705	18.1090	24.5967
G7	13.3502	14.4154	11.1004	13.1766	17.1790	15.4046	15.4410
G8	11.9997	10.1568	10.0931	12.7028	12.8659	15.5091	11.8493
G9	8.8130	12.7348	9.7482	9.4237	9.2001	19.2648	15.3030
G10	10.9483	12.9087	9.1469	12.9949	14.4919	15.7017	15.3854
G11	12.9223	11.2119	11.3103	13.4921	12.1474	11.7320	15.9133
G12	15.5356	13.8837	17.0493	17.4654	18.0373	12.1638	12.8343
G13	17.0283	17.1270	13.8930	18.3205	15.0348	16.2093	13.1365
G14	21.3208	21.0452	19.6949	22.4505	18.7570	20.6772	20.0283
G15	14.3129	14.8476	14.2646	16.1278	16.9950	14.0491	11.0995
G16	21.5027	23.8020	20.5059	22.7101	20.9788	18.7426	24.5884
G17	17.7687	18.6001	17.8334	15.7605	12.9234	19.2800	16.0935
G18	20.8535	19.3597	18.8672	20.7341	20.8799	19.0336	19.8678
G19	16.7841	15.7478	13.3738	20.1339	16.5294	17.8880	17.2921
G20	12.0318	8.9795	8.2954	14.4841	10.5924	8.1206	7.6505

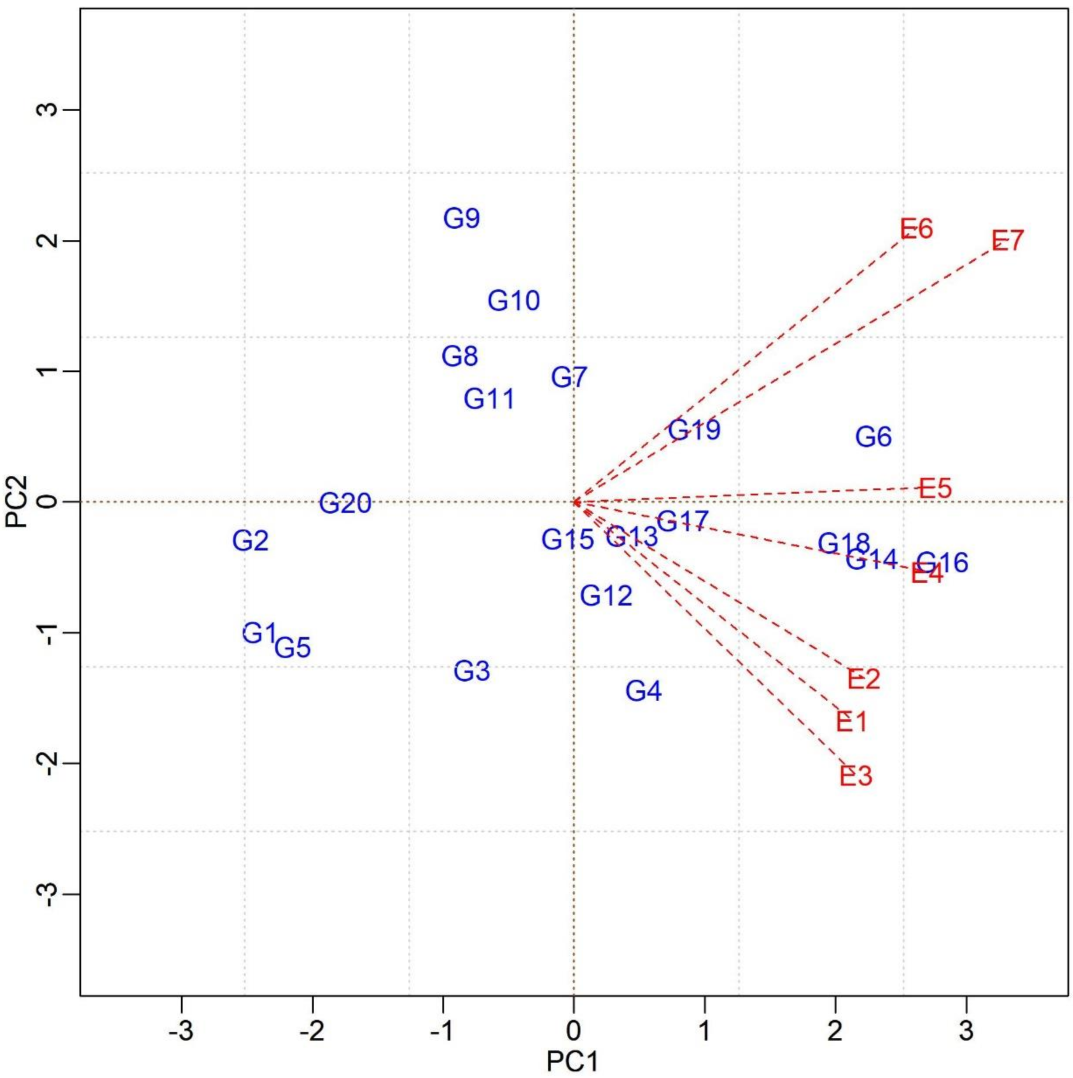

S1.1 Fig - Biplot representation of the data for the GGE model of fixed effects.
