# Supplementary material for: Shrinkage in the Bayesian analysis of the GGE model: A case study with simulation
Source: PLoS One. 2021 Aug 30;16(8):e0256882. doi: 10.1371/journal.pone.0256882 (PMC8405011; doi:10.1371/journal.pone.0256882)
Supplement: S2 Appendix — (PDF) [file pone.0256882.s008.pdf]

S2 Appendix

A priori derivation of the maximum entropy for $\sigma^2_{\lambda_k}$

Shannon entropy [1,2] for a continuous variable y is given by

$$H(y) = -\int_S p(\mathrm{y})\log(p(\mathrm{y}))\,\mathrm{dy} \tag{1}$$

In our framework, to derive maximum entropy density for $\sigma^2_{\lambda_k} \in (0,\infty)$, the only constraint assumed is that $E\left[\sigma^2_{\lambda_k}\right] = \vartheta\left(0 < \vartheta < \infty\right)$, and of course, density normalization for $p(\sigma^2_{\lambda_k})$. To make for easier algebraic identities we assumed $\tau_{\lambda_k} = 1/\sigma^2_{\lambda_k}$, that implies the following restrictions:

$$\int_0^\infty \tau_{\lambda_k} p(\tau_{\lambda_k}) d\tau_{\lambda_k} = \vartheta \Rightarrow \zeta\left[\int_0^\infty \tau_{\lambda_k} p(\tau_{\lambda_k}) d\tau_{\lambda_k} - \vartheta\right] \quad \text{and} \tag{2}$$

$$\int_0^\infty p(\tau_{\lambda_k}) d\tau_{\lambda_k} = 1 \Rightarrow \zeta_0\left[\int_0^\infty p(\tau_{\lambda_k}) d\tau_{\lambda_k} - 1\right] \tag{3}$$

Using Lagrange multipliers for the restrictions and maximizing over parameter space (takingfirst derivatives equal to zero) yields maximum entropy density distribution for $p(\tau_{\lambda_k})$ [2] :

$$p(\tau_{\lambda_k}) = \frac{\mathrm{e}^{-\varsigma\tau_{\lambda_k}}}{\int_0^\infty \mathrm{e}^{-\varsigma\tau_{\lambda_k}}\, d\tau_{\lambda_k}}. \tag{4}$$

Note that

$$\int_0^\infty \mathrm{e}^{-\varsigma\tau_{\lambda_k}}\, d\tau_{\lambda_k} = \int_0^\infty \left(\zeta/\zeta\right)\mathrm{e}^{-\varsigma\tau_{\lambda_k}}\, d\tau_{\lambda_k} = \left(1/\zeta\right)\int_0^\infty \zeta\,\mathrm{e}^{-\varsigma\tau_{\lambda_k}}\, d\tau_{\lambda_k} = 1/\zeta\,;\ \zeta > 0.$$

Then,

$$p(\tau_{\lambda_k}) = \mathrm{e}^{-\varsigma\tau_{\lambda_k}} \big/ (1/\varsigma) = \varsigma\,\mathrm{e}^{-\varsigma\tau_{\lambda_k}}\ .$$

The density of $\sigma^2_{\lambda_k}$, using linear transformation techniques [3] is given by:

$$p_{\sigma^2_{\lambda_k}}(\sigma^2_{\lambda_k}) = \left| \frac{\partial}{\partial \sigma^2_{\lambda_k}} g^{-1}(\sigma^2_{\lambda_k}) \right| p_{\tau_{\lambda_k}}\left(g^{-1}(\sigma^2_{\lambda_k}) \right) I_{D(\sigma^2_{\lambda_k})}, \qquad (5)$$

observing that $\sigma^2_{\lambda_k} = g(\tau_{\lambda_k}) = 1/\tau_{\lambda_k} \Rightarrow (\sigma^2_{\lambda_k})^{-1} = \tau_{\lambda_k}$. Solving the derivative within the modulus of equation (5) results in

$$\frac{\partial}{\partial \sigma^2_{\lambda_k}} g^{-1}(\sigma^2_{\lambda_k}) = \frac{\partial}{\partial \sigma^2_{\lambda_k}} \left(\frac{1}{\sigma^2_{\lambda_k}} \right) = -\frac{1}{(\sigma^2_{\lambda_k})^2}. \qquad (6)$$

Using (6) in (5) yields the maximum entropy density for $\sigma^2_{\lambda_k}$:

$$p(\sigma^2_{\lambda_k}) = \frac{\varsigma}{\Gamma(1)} (\sigma^2_{\lambda_k})^{-(1+1)} \mathrm{e}^{-\varsigma/\sigma^2_{\lambda_k}}$$

where $\Gamma(\cdot)$ is the gamma function. Therefore, $\sigma^2_{\lambda_k} \sim Inv - Gamma(a,b)$, with $\mathrm{a} = 1$ and $\mathrm{b} = \varsigma$.

References

1 Templeman AB, Xingsi LA. A maximum entropy approach to constrained non-linear programming. Engineering Optimization. 1987; 12(3): 191–205.

2 Cover TM, Thomas JA. Elements of information theory. 2ª ed. New Jersey: John Wiley & Sons; 1999.

3 Mood AM, Graybill FA, Boes, DC. Introduction to the theory of statistics. 3ª ed. Tokyo: McGraw-Hill; 1974.
