# Supplementary material for: Shrinkage in the Bayesian analysis of the GGE model: A case study with simulation
Source: PLoS One. 2021 Aug 30;16(8):e0256882. doi: 10.1371/journal.pone.0256882 (PMC8405011; doi:10.1371/journal.pone.0256882)
Supplement: S3 Appendix — (PDF) [file pone.0256882.s009.pdf]

S3 Apendix

Posterior distribution for variance components of $\left(\sigma^2_{\lambda_k}\right)$ singular values

Initially, the likelihood function is expressed as

$$L\left(\boldsymbol{\theta}, \sigma_e^2 | \, \mathbf{y}\right) = p(\mathbf{y} \,|\, \boldsymbol{\theta}, \sigma_e^2) = \frac{1}{(2\pi)^{\frac{n}{2}} \left|\mathbf{I}\sigma_e^2\right|^{\frac{1}{2}}} exp\left\{-\frac{1}{2\sigma_e^2}\left(\mathbf{y}-\boldsymbol{\theta}\right)^{\top}\left(\mathbf{y}-\boldsymbol{\theta}\right)\right\}. \quad (1)$$

The joint posterior distribution is then obtained by applying Bayes' theorem connecting *a priori* information with the likelihood function:

$$\begin{aligned} p\left(\boldsymbol{\Phi} | \, \mathbf{y}\right) = p(\mathbf{y} \,|\, \boldsymbol{\theta}, \sigma_e^2) \propto p(\boldsymbol{\beta} \,|\, \boldsymbol{\mu}_\beta, \sigma_\beta^2)\, p\left(\sigma_e^2 | v, u\right) \times \\ \times \prod_{k=1}^{t} p\left(\lambda_k | \mu_{\lambda_k}, \sigma^2_{\lambda_k}\right) p\left(\sigma^2_{\lambda_k}\right) p\left(\boldsymbol{\alpha}_{\boldsymbol{k}}\right) p\left(\boldsymbol{\gamma}_k\right) \end{aligned} \quad (2)$$

where $\boldsymbol{\Phi} = \left(\boldsymbol{\beta}, \boldsymbol{\alpha}, \boldsymbol{\gamma}, \boldsymbol{\lambda}, \sigma_e^2, \boldsymbol{\sigma}_\lambda\right)$, $\boldsymbol{\lambda} = (\lambda_1, \cdots, \lambda_t)$, $\boldsymbol{\sigma}_\lambda = (\sigma^2_{\lambda_1}, \cdots, \sigma^2_{\lambda_t})$ and $t = min(g-1, e)$.

Using prior distribution from maximum entropy reasoning $\left(\sigma^2_{\lambda_k} \sim GI(\mathrm{a}, b)\right)$, full conditional posterior density for $\sigma^2_{\lambda_k}$ is:

$$p\left(\sigma^2_{\lambda_k} | \cdots\right) \propto \left(\sigma^2_{\lambda_k}\right)^{-1} exp\left\{-\frac{1}{2\sigma^2_{\lambda_k}}\lambda_k^2\right\} \times \left(\sigma^2_{\lambda_k}\right)^{-a-1} exp\left\{-\frac{b}{\sigma^2_{\lambda_k}}\right\}, \quad (3)$$

$$\text{or } p\left(\sigma^2_{\lambda_k} | \cdots\right) \propto \left(\sigma^2_{\lambda_k}\right)^{-a-1-1} exp\left\{-\frac{1}{2\sigma^2_{\lambda_k}}\left(\lambda_k^2 + 2b\right)\right\} \text{ and} \quad (4)$$

$$p\left(\sigma^2_{\lambda_k} | \cdots\right) \propto \left(\sigma^2_{\lambda_k}\right)^{-(a+1)-1} exp\left\{-\frac{1}{2\sigma^2_{\lambda_k}}\left(\lambda_k^2 + 2b\right)\right\}. \quad (5)$$

That equation (5) represents an inverse gamma density with scale parameters $\left(\lambda_k^2 + 2b\right)$ and with $(a+1)$ degrees of freedom, or $\sigma^2_{\lambda_k} | \cdots \sim GI(a+1, \lambda_k^2 + 2b)$.
