# Supplementary material for: Shrinkage in the Bayesian analysis of the GGE model: A case study with simulation
Source: PLoS One. 2021 Aug 30;16(8):e0256882. doi: 10.1371/journal.pone.0256882 (PMC8405011; doi:10.1371/journal.pone.0256882)
Supplement: S4 Appendix — (PDF) [file pone.0256882.s010.pdf]

S4 Appendix

Exemplification of the MCMC sampling algorithm

The iterative sampling process for the BGGEE model was conducted from the following steps:

A1- Assign initial values to parameters: $\mathbf{\Phi}^0 = [\boldsymbol{\beta}^0, \lambda^0, \boldsymbol{\alpha}_k^0, \boldsymbol{\gamma}_k^0, (\sigma^2_{\lambda_k})^0, (\sigma^2_e)^0]$, with $k = 1, \cdots, t$.

A2- From these initial values, the *l*-th iteration can be obtained as follows:

a) Generate $\boldsymbol{\beta}^l \,|, \lambda^{l-1}, \boldsymbol{\alpha}^{l-1}, \boldsymbol{\gamma}^{l-1}, (\sigma^2_{\lambda_k})^{l-1}, (\sigma^2_e)^{l-1}$ from the a posteriori conditional (equation 4);

b) Generate the *l*-th observation of the bilinear parameters through the sequence d1), d2) and d3) below, for $k = 1, 2, ..., t$:

d1) Generate $\lambda_k^l \,|\, \boldsymbol{\alpha}^{l-1}, \boldsymbol{\gamma}^{l-1}, (\sigma^2_{\lambda_k})^{l-1}, (\sigma^2_e)^{l-1}, \boldsymbol{\beta}^l$ from the a posteriori conditional (equation 5);

d2) Generate $(\sigma^2_{\lambda_k})^l \,|\, \boldsymbol{\alpha}^{l-1}, \boldsymbol{\gamma}^{l-1}, (\sigma^2_e)^{l-1}, \boldsymbol{\beta}^l, \lambda_k^l$ from the a posteriori conditional (equation 6);

d3) Generate $(\boldsymbol{\alpha}_k)^l \,|\, \boldsymbol{\gamma}_k^{l-1}, (\sigma^2_e)^{l-1}, \boldsymbol{\beta}^l, \lambda_k^l, (\sigma^2_{\lambda_k})^l$:

i) Generate $\left(\tilde{\boldsymbol{\alpha}}_k\right)^l$ from the a posteriori conditional (equation 9);

ii) to obtain $\left(\boldsymbol{\alpha}_k\right)^l = \mathbf{H}_k \left(\tilde{\boldsymbol{\alpha}}_k\right)^l$.

d4) Generate $(\boldsymbol{\gamma}_k)^l \,|(\sigma^2_e)^{l-1}, \boldsymbol{\beta}^l, \lambda_k^l, (\sigma^2_{\lambda_k})^l, \boldsymbol{\alpha}_k^l$:

i) Generate $\left(\tilde{\boldsymbol{\gamma}}_k\right)^l$ from the a posteriori conditional (equation 10);

ii) to obtain $(\boldsymbol{\gamma}_k)^l = \mathbf{D}_k \left(\tilde{\boldsymbol{\gamma}}_k\right)^l$.

e) Generate $(\sigma^2_e)^l \,|\, \boldsymbol{\beta}^l, \lambda_k^l, \boldsymbol{\alpha}^l, \boldsymbol{\gamma}_k^l, (\sigma^2_{\lambda_k})^l$ from the a posteriori conditional (equation 11).

Return to A2 changing counter to $l+1$ and repeat this sequence of steps iteratively until convergence.

For the BGGE model, as illustrated in Oliveira et al. [35], just remove from the algorithm exemplified above, step d2), that is, the variance component $\sigma^2_{\lambda_k}$ is not sampled in the MCMC process and in equation (5) it is considered $\sigma^2_{\lambda_k} = 10^8$.
